# The chimeric ubiquitin ligase SH2-U-box inhibits the growth of imatinib-sensitive and resistant CML by targeting the native and T315I-mutant BCR-ABL

**DOI:** 10.1038/srep28352

**Published:** 2016-06-22

**Authors:** Yi Ru, Qinhao Wang, Xiping Liu, Mei Zhang, Daixing Zhong, Mingxiang Ye, Yuanchun Li, Hua Han, Libo Yao, Xia Li

**Affiliations:** 1 State Key Laboratory of Cancer Biology, Department of Biochemistry and Molecular Biology, The Fourth Military Medical University, Xi’an, Shaanxi 710032, China; 2 Department of Biochemistry and Molecular Biology, Zunyi Medical College, Zunyi, Guizhou 563000, China; 3 Department of Thoracic Surgery, Tangdu Hospital, the Fourth Military Medical University, Xi’an, Shaanxi 710038, China; 4 Department of Pulmonary Medicine, Xijing Hospital, The Fourth Military Medical University, Xi’an, Shaanxi 710032, China; 5 Department of Hematology, Tangdu Hospital, The Fourth Military Medical University, Xi’an, Shaanxi 710038, China

## Abstract

Chronic myeloid leukemia (CML) is characterized by constitutively active fusion protein tyrosine kinase BCR-ABL. Although the tyrosine kinase inhibitor (TKI) against BCR-ABL, imatinib, is the first-line therapy for CML, acquired resistance almost inevitably emerges. The underlying mechanism are point mutations within the *BCR*-*ABL* gene, among which T315I is notorious because it resists to almost all currently available inhibitors. Here we took use of a previously generated chimeric ubiquitin ligase, SH2-U-box, in which SH2 from the adaptor protein Grb2 acts as a binding domain for activated BCR-ABL, while U-box from CHIP functions as an E3 ubiquitin ligase domain, so as to target the ubiquitination and degradation of both native and T315I-mutant BCR-ABL. As such, SH2-U-box significantly inhibited proliferation and induced apoptosis in CML cells harboring either the wild-type or T315I-mutant BCR-ABL (K562 or K562R), with BCR-ABL-dependent signaling pathways being repressed. Moreover, SH2-U-box worked in concert with imatinib in K562 cells. Importantly, SH2-U-box-carrying lentivirus could markedly suppress the growth of K562-xenografts in nude mice or K562R-xenografts in SCID mice, as well as that of primary CML cells. Collectively, by degrading the native and T315I-mutant BCR-ABL, the chimeric ubiquitin ligase SH2-U-box may serve as a potential therapy for both imatinib-sensitive and resistant CML.

Chronic myeloid leukemia (CML) is a myeloproliferative clonal disorder disease characterized by the cytogenetic hallmark of Philadelphia (Ph) chromosome, which exists in >90% of CML patients and is caused by reciprocal translocation of the t(9;22)(q34;q11). Fusion of Abelson (*ABL*) tyrosine kinase gene on chromosome 9 with break-point cluster region (*BCR*) gene on chromosome 22 creates the *BCR-ABL* oncogene, leading to the constitutively active tyrosine kinase BCR-ABL^1^,^2^,^3^. By recruiting the adaptor proteins such as Grb2^4^,^5^ and CrkL^6^,^7^, BCR-ABL activates several signaling pathways, including PI3K-Akt, MAPK^4^,^8^,^9^,^10^ and STAT5^11^, thus leading to uncontrolled cell proliferation and CML pathogenesis^8^,^12^. Therefore, targeting BCR-ABL, the key player and “addiction” oncogene of CML, has been a vital strategy for CML therapy^13^.

Imatinib mesylate (IM, also known as Gleevec or STI-571), which is the first FDA approved tyrosine kinase inhibitor (TKI) that competitively binds to the ATP-binding site of BCR-ABL, has shown remarkable clinical activity for newly diagnosed CML^14,15^. Despite its impressive success, imatinib-resistance has emerged as a prominent clinical problem in CML treatment. The most important mechanism for IM-resistance is point mutations occurring at more than 40 different amino acid positions within BCR-ABL kinase domain^16^. Although the second generation TKIs (dasatinib, nilotinib and INNO-406) and the third generation TKI (bosutinib) have been developed and improved the treatment outcome^17^,^18^, they were unable to overcome resistance caused by the gatekeeper mutation T315I^18^,^19^,^20^,^21^, which accounts for approximately 20% acquired resistance cases^22^. Ponatinib holds the promise because it is a potent inhibitor not only for native BCR-ABL but also for all known BCR-ABL mutants, including BCR-ABL T315I^23^, however, its adverse effect including myelosuppression and pancreatitis limits its wide use^24^. Hence, BCR-ABL-dependent resistance remains to be a major challenge in the field and novel strategies are still required in CML therapy.

Ubiquitination and degradation of proteins has been implicated as a main route for regulating intracellular signals in eukaryotic cells. Generally, proteins tagged for proteasomal degradation are covalently modified by a polyubiquitin chain, which consists of more than four ubiquitin moiety and serves as a signal of proteolytic destruction. The ubiquitination process is carried out by a cascade of enzymatic reactions involving E1 (ubiquitin-activating enzyme), E2 (ubiquitin-conjugating enzyme) and E3 (ubiquitin ligase)^25^. Among them, E3s mainly confer substrate specificity because they are flexible in structure and responsible for interacting with and mediating the transfer of ubiquitin to the substrates^26^. According to the functional domains, E3s can be divided into three major classes: HECT (homologous to the E6-AP carboxyl terminus) E3^27^, RING finger E3^28^, and U-box E3^29^. Cbl belongs to a RING finger E3 and has been implicated in the negative regulation of various protein tyrosine kinases^30^. The U-box protein CHIP (carboxy terminus of Hsc70 interacting protein) can bind to the molecular chaperone Hsc70 and Hsp90 and mediates ubiquitination and subsequent degradation of their client proteins^31^. Previously, we successfully generated several chimeric ubiquitin ligases by fusing RING domain of Cbl or U-box domain of CHIP with a protein-protein interaction domain to specifically target the oncoproteins such as HER2^32^, EGFR^33^ and IR/IGF-1R^34^, and we also demonstrated that they could effectively inhibited these oncoproteins-related tumors.

Both wild type and BCR-ABL T315I lead to CML pathogenesis via signal transduction, which is initiated by binding of phosphorylation motif of BCR-ABL with the adapter proteins, for example, SH2 domain-containing protein Grb2^35^, consequently resulting in activation of the downstream signaling pathway. In the present study, we utilized a previously created chimeric ubiquitin ligase, SH2-U-box, which harbors SH2 domain of Grb2 and U-box domain of CHIP, to target both the wild type and mutant BCR-ABL and rewire them to degradation pathway. We first checked whether SH2-U-box is able to promote ubiquitination and degradation of BCR-ABL and BCR-ABL T315I, and then examined its effect on imatinib-sensitive K562 cells and imatinib-resistant, BCR-ABL T315I-harboring K562R cells. Finally, we evaluated the effect of SH2-U-box-carrying lentivirus on the growth of K562-xenografts in nude mice, K562R-xenografts in Severe Combined Immune-deficiency (SCID) mice, as well as primary CML cells.

## Results

### The chimeric ubiquitin ligase SH2-U-box downregulates BCR-ABL and BCR-ABL T315I in protein level

**Figure 1:**
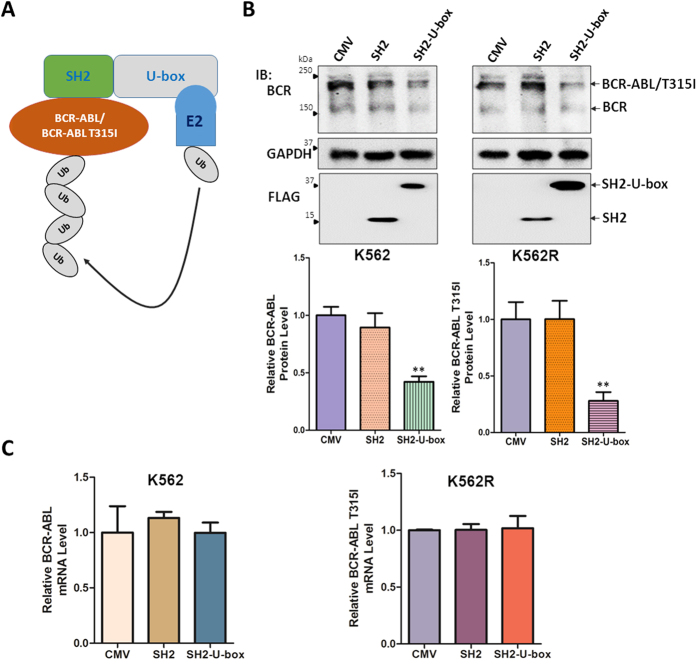
The chimeric ubiquitin ligase SH2-U-box downregulates BCR-ABL and BCR-ABL T315I in protein level. (**A**) Schematic diagram of SH2-U-box to target BCR-ABL or BCR-ABL T315I. (**B,C**) K562 and K562R cells were transiently electroporated with pFLAG-CMV-4 empty vector (CMV), pFLAG-CMV-4-SH2 (SH2) or pFLAG-CMV-4-SH2-U-box (SH2-U-box). BCR-ABL/BCR-ABL T315I protein levels were analyzed by Western blotting, the relative band intensity of BCR-ABL was derived from three independent experiments and presented as a bar graph (n = 3) (**B**) and their mRNA levels were measured by quantitative real-time PCR (n = 3) (**C**). **p < 0.01, Student’s *t* test.

The chimeric ubiquitin ligase SH2-U-box was generated previously^33^ and we demonstrated that it can mediate down-regulation of EGFR in Non-Small Cell Lung Cancer (NSCLC). Besides EGFR, the SH2 domain from Grb2 can also recognize and bind to the phosporylation sites of BCR-ABL or BCR-ABL T315I^36^. The CHIP U-box domain of SH2-U-box confers E3 activity and is able to transfer E2-loaded ubiquitin to the target protein bound via SH2 domain (Fig. 1A). Therefore, we sought to determine whether SH2-U-box can be utilized to target the degradation of native and T315I-mutant BCR-ABL.

To this end, pFLAG-CMV-4 empty vector (CMV), pFLAG-CMV-4-SH2-U-box (SH2-U-box), as well as pFLAG-CMV-4-SH2 (SH2), which encodes only SH2 domain of Grb2, were respectively electroporated into K562 or imatinib-resistant K562R cells, and then the wild type or mutant BCR-ABL expression levels were assessed. As shown in Fig. 1B, SH2-U-box was able to remarkably decrease both BCR-ABL and BCR-ABL T315I in protein levels relative to CMV and SH2. We further confirmed that the downregulation of BCR-ABL and BCR-ABL T315I were post-transcriptional events because their mRNA levels measured by quantitative real-time PCR were not changed significantly (Fig. 1C). Thus, SH2-U-box is able to downregulate native as well as T315I-mutant BCR-ABL in protein level.

### The chimeric ubiquitin ligase SH2-U-box interacts with BCR-ABL or BCR-ABL T315I and promotes their ubiquitination and degradation

**Figure 2:**
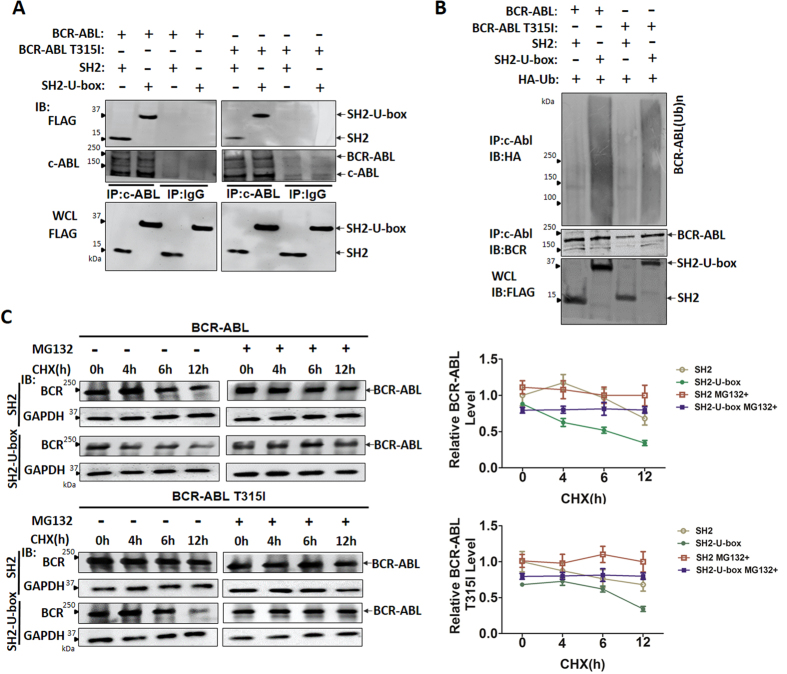
SH2-U-box interacts with BCR-ABL or BCR-ABL T315I and promotes their ubiquitination and degradation. (**A**) 293 T cells were transfected with the indicated constructs and the interaction between SH2-U-box and BCR-ABL or BCR-ABL T315I were determined by co-immunoprecipitation assay. (**B**) 293T cells were transfected with the indicated constructs together with pcDNA3.1(+)-3 × HA-Ub and treated with MG-132 (10 μM) for 4–6 hours. BCR-ABL/BCR-ABL T315I ubiquitination were assessed by *in vivo* ubiquitination assay as described in Methods. Whole cell lysates (WCL) were subjected to Western blotting with an anti-FLAG. (**C**) 293T cells transfected as indicated were treated with CHX (50 μg/ml) for 0, 4, 6 and 12 h in the absence or presence of MG132 (10 μM). BCR-ABL/BCR-ABL T315I protein stability was analyzed by Western blotting and quantified as relative band intensity from three independent experiments (n = 3).

Next, we checked whether SH2-U-box can interact with and promote the ubiquitination of BCR-ABL or BCR-ABL T315I as we expected. To this end, FLAG-tagged SH2 or SH2-U-box was co-transfected with pcDNA3.1(−)-BCR-ABL or pcDNA3.1(−)-BCR-ABL T315I into 293T cells respectively, and then co-immunoprecipitation assay was performed. As shown in Fig. 2A, SH2-U-box existed in the immunoprecipitation complex of BCR-ABL as did SH2, indicating that SH2-U-box interacted with BCR-ABL and BCR-ABL T315I as SH2 did. *In vivo* ubiquitination assay demonstrated that SH2-U-box markedly enhanced the ubiquitination of both BCR-ABL and BCR-ABL T315I when compared with SH2 (Supplementary Fig. 1 and Fig. 2B). We further compared the stability of ectopically expressed BCR-ABL and BCR-ABL T315I in SH2- and SH2-U-box-transfected 293T cells by conducting CHX chase experiment. It was shown that either wild type or T315I mutant BCR-ABL in SH2-U-box group was more unstable than that in SH2 group, but such phenomenon was almost abrogated by proteasome inhibitor MG132 (Fig. 2C), confirming that SH2-U-box enhanced the degradation of BCR-ABL and BCR-ABL T315I in a proteasome-dependent manner. Together, these results indicated that SH2-U-box is able to associate with the wild type and T315I-mutant BCR-ABL, promoting their ubiquitination and proteasomal degradation.

### SH2-U-box inhibits the growth of K562 and K562R cells and exerts additive effect with imatinib in K562 cells

**Figure 3:**
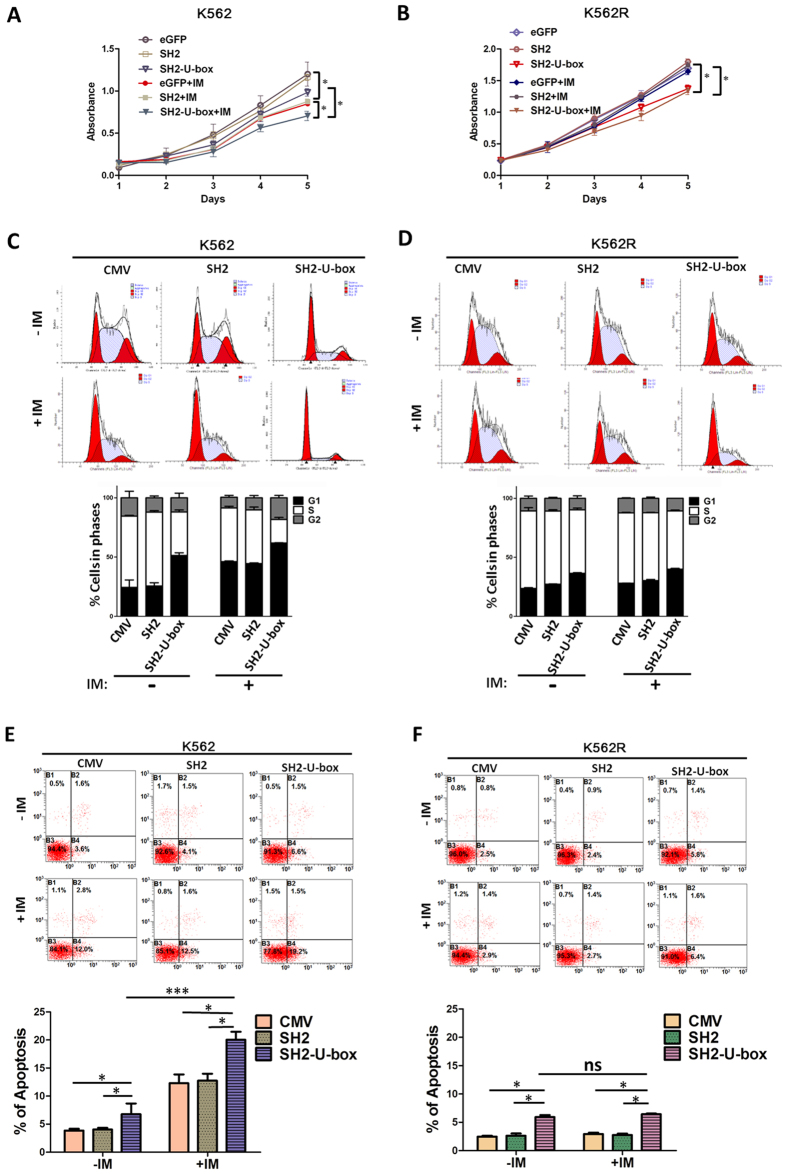
SH2-U-box inhibits the growth of K562 and K562R cells and exerts additive effect with imatinib in K562 cells. (**A,B**) K562 and K562R cells stably expressing eGFP, SH2 and SH2-U-box were incubated with or without 0.5 μM imatinib for 5 days. Cell growth were measured by CCK-8 assay (n = 6). (**C–F**) K562 and K562R cells were transiently electropored with CMV, SH2- or SH2-U-box-encoding plasmids and 24 hours later, incubated with or without 0.5 μM imatinib for 24 hours. Cell cycle was examined by flow cytometric analysis (n = 3) (**C,D**) and cell apoptosis was determined by PI-Annexin V staining followed by flow cytometric analysis (n = 3)(**E,F**). Each experiment was repeated at least three times independently and the representative results are the mean ± S.D. from six-replicates (**A,B**) or triplicate (**C–F**). *p < 0.05, ***p < 0.001, Student’s *t* test.

Given that the survival and proliferation of CML cells were “addicted” to the constitutively active BCR-ABL^37^,^38^, we asked whether SH2-U-box-caused downregulation of BCR-ABL or T315I mutant can inhibit K562 or K562R or affect cells’ response to imatinib, the first line therapy of CML. For this reason, K562 and K562R cells stably expressing eGFP, SH2 or SH2-U-box, which were generated via lentivirus infection with pLenti6.3-IRES2-EGFP, pLenti6.3-SH2-IRES2-EGFP or pLenti6.3-SH2-U-box-IRES2-EGFP (Supplementary Fig. 2), were treated with or without imatinib and subjected to CCK-8 assay. As shown in Fig. 3A,B, SH2-U-box, but not SH2, significantly inhibited cell proliferation in both K562 and K562R cells when compared with eGFP. Furthermore, expression of SH2-U-box, other than SH2, acted in concert with imatinib to inhibit cell proliferation in imatinib-sensitive K562 cells (Fig. 3A) but not imatinib-resistant K562R cells (Fig. 3B).

To further confirm the suppressive effect of SH2-U-box on cell proliferation, flow cytometry assay were performed to profile cell cycle distribution. K562 and K562R cells transiently electroporated with pFLAG-CMV-4, pFLAG-CMV-4-SH2 and pFLAG-CMV-4-SH2-U-box were analyzed and the results showed that SH2 -U-box expression in K562 led to a significant increase in the proportion of cells in G1 phase (51.4 ± 2.2% *v.s.* 24.4 ± 6.2% and 25.6 ± 2.7% for CMV and SH2, p = 0.03 and 0.009 respectively), concomitant with a decrease in S phase proportion (36.7 ± 1.7% *v.s.* 60 ± 0.7% and 62.3 ± 1.2% for CMV and SH2, p = 0.003 and 0.003 respectively; Fig. 3C). Similar results were observed in K562R cells (23.6 ± 0.8%, 27.2 ± 0.2% and 36.4 ± 0.6% of G1 proportion and 65.7 ± 2.7%, 65 ± 1.1% and 53.8 ± 1.5% of S proportion for CMV, SH2 and SH2-U-box, respectively; Fig. 3D). However, only in K562 cells did SH2 -U-box act additively with imatinib to inhibit cell cycle progression, evidence by more cell arrest in G1 phase in SH2 -U-box plus imatinib group when compared with CMV plus imatinib group and SH2 -U-box group (61.9 ± 0.2% *v.s.* 46.2 ± 0.6% and 51.4 ± 2.2%, p = 0.0008 and 0.02, respectively; Fig. 3C), but such additive effect was not observed in K562R cells (Fig. 3D).

Next, we addressed whether SH2 -U-box could induce cell apoptosis or enhance imatinib-induced apoptosis. Apoptotic cells were determined by PI-Annexin V double staining followed by flow cytometry analysis. Indeed, the expression of SH2-U-box was able to increase apoptosis in both K562 (6.5 ± 1% *v.s.* 3.5 ± 0.6%, p = 0.02; Fig. 3E) and K562R cells (5.8 ± 0.6% *v.s*. 2.5 ± 0.7%, p = 0.001; Fig. 3F), whereas the expression of SH2 had no such effect in both cell type. The cell apoptosis were further confirmed by PARP cleavage in SH2-U-box-treated K562 and K562R cells (Supplementary Fig. 3). Moreover, SH2-U-box-expression rendered K562 cells more sensitive to imatinib-induced apoptosis, as indicated by more apoptotic cells in SH2-U-box group compared with CMV group upon imatinib treatment (20 ± 1.4% *v.s.* 12.3 ± 1.6%, p = 0.0001; Fig. 3E). However, CMV-, SH2- or SH2-U-box-transfected K562R cells were resistant to imatinib-induced apoptosis, which was evinced by no significant increase in cell apoptosis upon imatinib treatment (Fig. 3F). Collectively, these results indicated that dowregulation of native and T315I-mutant BCR-ABL by SH2-U-box inhibits the growth of both K562 and K562R via proliferation-inhibition and apoptosis-induction. SH2-U-box also exerts additive effect with imatinib in K562 cells.

### SH2-U-box inhibits BCR-ABL-mediated signaling in K562 and K562R cells

**Figure 4:**
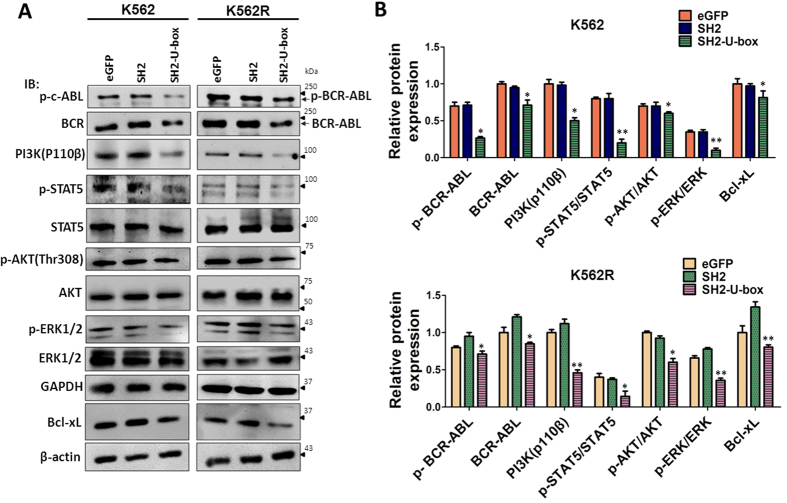
SH2-U-box inhibits BCR-ABL-dependent signaling in K562 and K562R cells. K562 and K562R stably expressing eGFP, SH2 and SH2-U-box were subjected to Western blotting, with phosphorylation of BCR-ABL/BCR-ABL T315I and downstream STAT5/Bcl-xL, PI3K/Akt and MAPK signaling being detected. The relative band intensity was derived from three independent experiments and presented as a bar graph (n = 3). *p < 0.05, **p < 0.01, Student’s *t* test.

The malignant characteristic of CML, such as sustaining proliferation and apoptosis-resistance, attributes to the constitutively active BCR-ABL, which phosphorylates its substrates and activates several downstream signal pathways, including STAT5, MAPK and PI3K-Akt^39^. The degradation of the native and mutant BCR-ABL by SH2-U-box and its inhibitory effect on K562 and K562R cells encouraged us to examine the signaling profile in these cells. As shown in Fig. 4A,B, SH2-U-box potently decreased the total and phosphorylated BCR-ABL. Furthermore, phosphorylated rather than total STAT5, an important downstream target of BCR-ABL^40^, as well as its target Bcl-xL, were also reduced by SH2-U-box in both K562 and K562R cells. The common pro-survival pathway, PI3K-Akt and MAPK were suppressed by SH2-U-box too. These data suggested that SH2-U-box-caused inhibition in the activation of BCR-ABL/BCR-ABL T315I and the downstream signaling pathways contributes to its inhibitory effect on K562 and K562R cells.

### SH2-U-box suppresses the growth of K562- and K562R- xenograft

**Figure 5:**
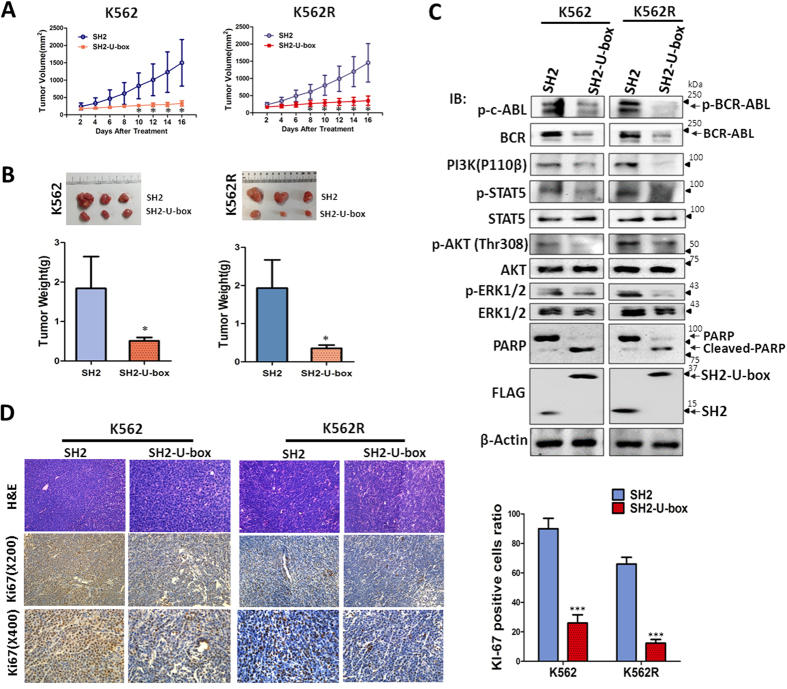
SH2-U-box suppresses the growth of K562- and K562R- xenograft. Subcutaneous K562- and K562R-xenografts were established by injecting 1 × 10^7^ cells into nude mice or SCID mice at both flanks and thereafter, each mouse was treated with SH2-carry lentivirus at one side and SH2-U-box-carrying lentivirus at the other side. (**A**) Tumors growth were measured (n = 3). (**B**) At the end of the experiment, tumors were removed and weighed (n = 3). (**C**) The tumor samples were prepared and subjected to Western blotting with BCR-ABL signaling antibodies, PARP and FLAG antibody. (**D**) H&E staining and Ki-67 immunostaining analysis were performed with the tumor tissues. The percentage of Ki-67 positive cells was calculated and presented as bar graph (n = 3). *p < 0.05, ***p < 0.001, Student’s *t* test.

We next examined whether SH2-U-box could suppress the *in vivo* growth of K562- and K562R-xenograft in mouse model. Because it was difficult for K562R cells to form the tumor in nude mice, we subcutaneously established K562-xenografts in nude mice and K562R-xenografts in SCID mice, respectively. Each mouse bore the tumors at both flanks and was treated with pLenti-SH2 at one side and pLenti-SH2-U-box at the other side via intratumoral injection of the lentivirus. As shown in Fig. 5A, the tumors at the sides of SH2-U-box-treatment grew much slower than those at the sides of SH2-treatment for both K562 and K562R. As a result, the average weight of the xenografts treated with SH2-U-box were significantly less than those treated with SH2, either for K562 (510 mg ± 85 mg *v.s.* 1840 mg ± 805 mg, p = 0.046), or for K562R (350 ± 85 mg *v.s.* 1900 ± 740 mg, p = 0.021. Fig. 5B).

To further confirm the *in vivo* effect of SH2-U-box, we analyzed the BCR-ABL signaling pathway in the tumor tissues derived from the xenografts. Consistent with *in vitro* studies, the expression levels of pBCR-ABL, pSTAT5, PI3K(p110β), pAkt and pERK were decreased (Fig. 5C). Immunostaining assay (Fig. 5D) also showed that the number of Ki67-positive cells in the tumor derived from SH2-U-box-treated groups was markedly decreased compared with SH2-treated group. It was also shown that SH2-U-box treatment led to cell apoptosis *in vivo*, which was evinced by cleaved PARP (Fig. 5C). These results reinforced SH2-U-box’s inhibitory effect on BCR-ABL signaling and cell growth, implying its potential efficacy as a therapeutic agent.

### SH2-U-box exert inhibitory role in primary CML cells

**Figure 6:**
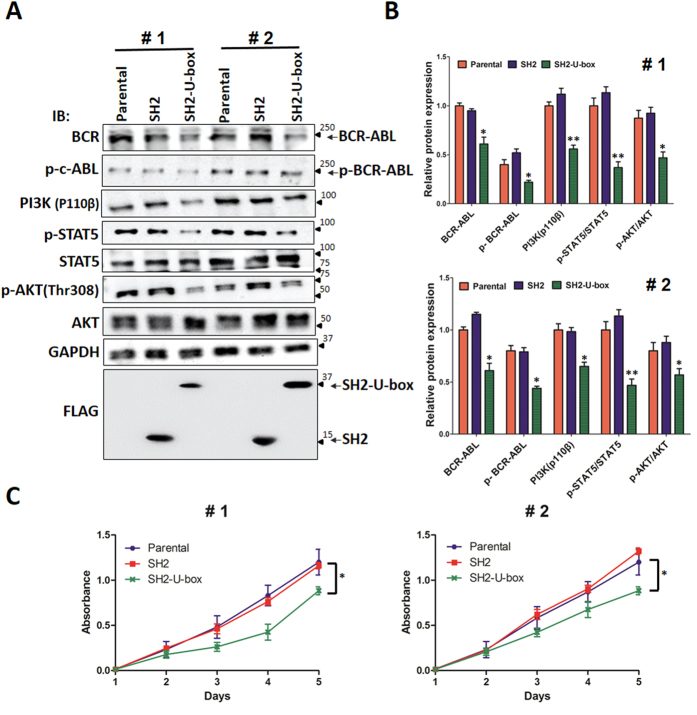
SH2-U-box suppresses primary CML cells. The primary CML cells were infected with SH2- or SH2-U-box-carrying lentivirus and subjected to Western blotting and CCK-8 assay. (**A,B**) BCR-ABL, p-BCR-ABL and downstream STAT5/Bcl-xL, PI3K/Akt and MAPK signaling were detected 48 hours post infection, with the relative band intensity derived from three independent experiments presented as a bar graph (n = 3). (**C**) Cell growth were measured by CCK-8 assay. The experiment was repeated three times independently and the representative result is the mean ± SD from six-replicates. *p < 0.05, **p < 0.01, Student’s *t* test.

Finally, we evaluated the activity of SH2-U-box in primary CML cells. Bone marrow mononuclear and granulocytes from 2 CML patients were isolated and infected with pLenti6.3-SH2-IRES2-EGFP or pLenti6.3-SH2-U-box-IRES2-EGFP and then BCR-ABL signaling pathway and cell growth were examined. As shown in Fig. 6A,B, SH2-U-box potently decreased BCR-ABL, p-BCR-ABL, p-STAT5/STAT5, PI3K and p-Akt/Akt. CCK-8 assay further demonstrated that SH2-U-box, but not SH2, substantially inhibited cell proliferation (Fig. 6C). These data suggested that SH2-U-box-caused inhibition in BCR-ABL and its downstream signaling pathways leads to growth inhibition in primary CML cells.

## Discussion

Ubiquitin-dependent protein degradation is a major way to eliminate the proteins that are misfolded or tightly regulated in cells. Specific degradation of a protein by harnessing the endogenous ubiquitin-proteasome system is an alternative and novel approach to knock down the intended target protein at protein levels, also known as “protein knockout^41^,^42^,^43^”. One of such approach is a chemical Proteolysis Targeting Chimera (PROTAC), in which a heterobifunctional molecule recruits a specific protein target to an E3 ubiquitin ligase, resulting in the target’s ubiquitination and degradation^41^,^44^,^45^. This technology has been actively employed to degrade the oncoproteins, such as ERα, AR^46^, BRD4^47^, as well as BCR-ABL^48^. Another approach is to create a non-endogenous, chimeric E3 ubiquitin ligase that can specifically target the proteins of interest, by replacing the “recognizing and binding” region of an endogenous E3 by an adaptor molecule or directly fusing an E3 “catalytic” domain with an interacting domain of a specific adaptor. With these chimeric E3s, we and others successfully rewired the oncoproteins such as Myc^49^, KRAS^50^, HER2^32^,^42^, EGFR^33^,^42^, and IR/IGF-1R^34^ to ubiquitin-proteasome degradation route, and as a result, the oncogenes-related malignant behaviors of tumor cells as well as *in vivo* tumor growth were remarkably inhibited. These promising results prompted us to test whether such a protein knockout strategy could be applied to targeted degradation of the wild type and mutant BCR-ABL in CML.

It has been well established that SH2-containing adapter protein Grb2 binds directly to the phosphorylated tyrosine 177 of BCR-ABL and activates MAPK pathway and PI3K/Akt pathway in CML^35^,^51^. Importantly, such a binding between Grb2 SH2 domain and BCR tyrosine 177 is required for induction of CML-like myeloproliferative disease driven by BCR-ABL in mouse model^35^. Therefore, in this study we hijacked such a binding ability of Grb2 SH2 and used a previously generated chimeric E3, SH2-U-box, in which SH2 domain of Grb2 serves as an “binding domain” and U-box domain from CHIP functions as E3 catalytic domain^52^. In principle, this chimeric E3 will be able to bind to Tyr 177 of BCR part through its SH2 domain, label ubiquitin tag to BCR-ABL fusion protein through its U-box domain, regardless of whether there is a mutation in ABL kinase domain, and subsequently lead to its degradation. Indeed, our results clearly demonstrated that SH2-U-box can directly bind to BCR-ABL/BCR-ABL T315I and lead to their ubiquitination and proteolysis. As a result, the downstream effectors including PIK3/Akt, MAPK and p-STAT5 were repressed and *in vitro* and *in vivo* growth of both imatinib-sensitive and resistant CML cells, i.e., BCR-ABL-harboring K562 and BCR-ABL T315I-harboring K562R were inhibited. More importantly, SH2-U-box exerts inhibitory influence on primary CML cells. Thus, our results convincingly support the notion that knocking down/out the “addiction” oncoproteins by UPS is an alternative and effective method for CML and other cancer treatment.

A prominent advantage of this chimeric SH2-U-box is that it can target the molecules that account for CML pathogenesis and imatinib-resistance simultaneously. Although we only examined native and T315I-mutant BCR-ABL in this study, we speculate that SH2-U-box can also target the degradation of other mutant forms of BCR-ABL, as long as the phosphorylation of BCR part and its binding with Grb2 SH2 occurs in the process of signal transduction. Moreover, because this “protein knockout” strategy eliminates the disease-causing proteins directly in protein level, its potential clinical use may prevent the occurrence of acquired resistance like TKI-induced secondary mutations of tyrosine kinases, which is the main mechanisms for drug resistance. Most recently, BCR-ABL compound mutations, defined as a BCR-ABL allele harboring two or more mutations (including T315I or not), have been reported to confer resistance to all available TKI, including ponatinib, the only approved TKI effective for all BCR-ABL single mutants^53^. Given its mechanism mentioned above, our chimeric SH2-U-box will also be able to target these compound mutations and thus even overcome ponatinib-resistance, which merits further study in the future.

The chimeric SH2-U-box exerts anti-CML function through degrading its intend targets, thus its mechanism is different from but complementary to that of tyrosine inhibitors. This suggests that the combination of these two strategies is reasonable. Particularly when eradication of active BCR-ABL by SH2-U-box is incomplete, the rest can be inhibited by the kinase inhibitors. Indeed, our results demonstrated that combination of SH2-U-box and imatinib exerts more profound effects as for proliferation inhibition and apoptosis induction than either alone in K562 cells. Of note, this notion is strongly supported by the finding that imatinib works in concert with the drug whose mechanism involves the degradation of BCR-ABL. For example, arsenic, a well known curative agent for acute promyelocytic leukemia, was shown to induce degradation of BCR-ABL by directly binding an E3, c-Cbl, and preventing its self-uiquitination/degradation^54^. As such, arsenic acts additively with imatinib to arrest cell cycle, induce apoptosis of CML cells and increase survival time of CML mice^55^. Thus, therapeutic application of BCR-ABL-degrading agent in combination with tyrosine kinase inhibitor will benefits more CML patients than either alone.

We finally tested the efficacy of SH2-U-box-carrying lentivirus in subcutaneous K562- or K562R-xenografts in nude or SCID mice and in primary CML cells. Consistent with the *in vitro* results in cell lines, SH2-U-box downregulated BCR-ABL-dependent downstream signaling and inhibits cell growth in K562- or K562R-xenografts, as well as primary CML cells. Interestingly, the *in vivo* activity of SH2-U-box seems to be more potent than that *in vitro*, probably because the xenografts received repetitious pLenti-SH2-U-box treatment. In addition, we noticed that SH2-U-box is not able to completely remove oncogenic Bcr-Abl or Bcr-Abl T315I from CML cells lines and primary CML cells. It is possible that the amount or the conformational structure of SH2-U-box is still not optimal for BCR-ABL degradation. Most recently, Lai *et al*.^48^ screened several PROTACs that were designed for the degradation of BCR-ABL/c-ABL and found only some of them works, where the degradation profiles were determined not only by the “ligand” for BCR-ABL/c-ABL, but also by the recruited E3 ligase. Actually, we had also tested another previously generated chimeric E3, SH2-RING^33^, in which RING domain is from Cbl ubiquitin ligase, and found its efficacy against BCR-ABL is weaker than SH2-U-box (data not shown). Therefore, the future study is warranted to improve the structure of such therapeutic chimeric E3 and optimize the delivery method and dosage.

**Figure 7:**
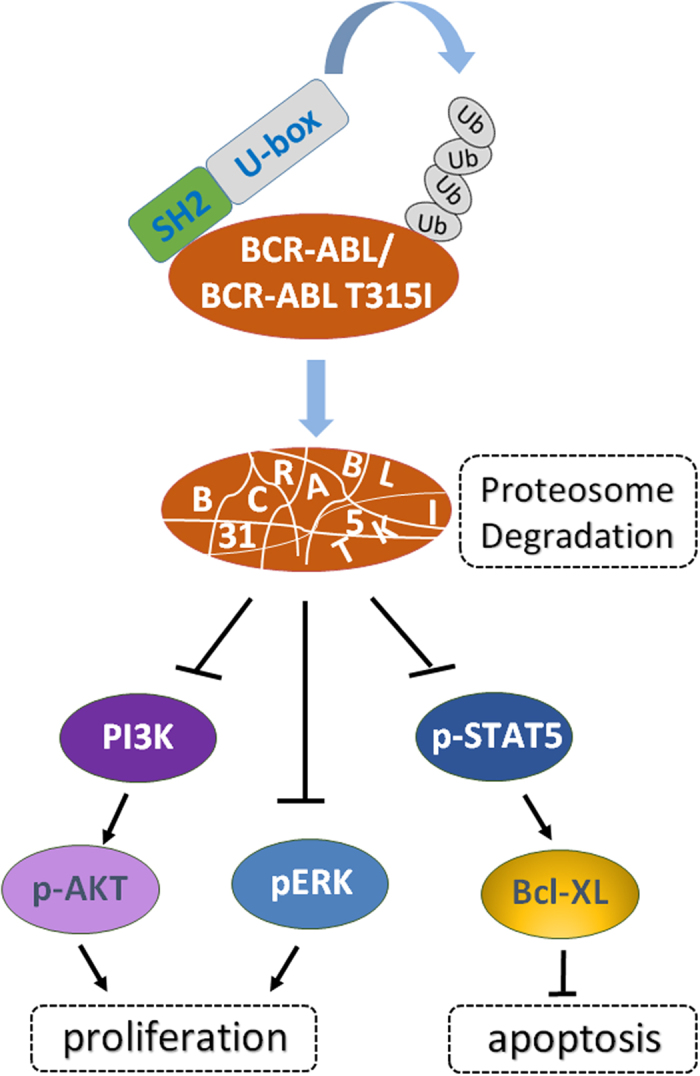
Working model of SH2-U-box. The chimeric ubiquitin ligase SH2-U-box is able to interact with BCR-ABL and BCR-ABL T315I, promoting their ubiquitination and subsequent degradation. As a result, SH2-U-box attenuates BCR-ABL-dependent pathway, inhibits the proliferation and induces the apoptosis of imatinib- sensitive and resistant CML cells.

In summary, we provided a chimeric ubiquitin ligase, SH2-U-box, which can target both the wide type and BCR-ABL T315I that exist in imatinib-sensitive or -resistant CML. By promoting the ubiquitination and degradation of the native or mutant BCR-ABL, SH2-U-box blocked BCR-ABL-dependent signaling pathway, and thus inhibited cell proliferation and induced cell apoptosis in both imatinib-sensitive and resistant CML cells (Fig. 7). Moreover, SH2-U-box works in concert with imatinib in imatinib-sensitive CML and it is efficient in primary CML cells. Thus, our study not only provide an alternative therapeutic strategy for overcoming imatinib-resistance, but also brought the light for combination therapy to improve CML treatment.

## Methods

### Cell lines and primary CML cells

BCR-ABL-harboring human chronic myeloid leukemia cell lines K562 were from ATCC and maintained in RPMI 1640 supplemented with 10% fetal bovine serum (Life Technologies, Inc.). Imatinib-resistant, BCR-ABL T315I-harboring K562R which were derived from K562 were established and provided by Beijing Cancer Hosipital, China. Briefly, K562 cells were cultured in RPMI 1640 supplemented with 10% fetal bovine serum in the presence of increasing concentration of imatinib (from 10 nM to 10 μM). The obtained single clone which harbors BCR-ABL T315I were named as K562R and routinely maintained in the same medium with 100 nM imatinib. Human embryonic kidney 293 T cells were maintained in DMEM supplemented with 10% fetal bovine serum. 100 μg/mL streptomycin and 100 U/mL penicillin were routinely added in the medium. Bone marrow of CML patients were obtained from discarded material utilized for routine laboratory tests at the Department of Hematology, Tangdu Hospital. The use of these materials is approved by the Fourth Military Medical University medical ethics committee with the informed consent obtained from the patients. Mononuclear cells and granulocytes were isolated by Histopaque gradient centrifugation (density 1.077; Sigma-Aldrich). Contaminating red cells were lysed in 0.8% ammonium chloride solution for 10 min. Isolated cells were suspended in RPMI 1640 supplemented with 15% FCS.

### Plasmid construction and cell transfection

SH2 and SH2-U-box were cloned into pFLAG-CMV-4 expression vector as previously described^33^. pGD210 (*BCR-ABL*) plasmid was kindly provided by Department of Hematology, Tangdu hospital, the Fourth Military Medical University. The N- and C-terminal fragments of BCR-ABL was amplified from pGD210 plasmid by piecewise PCR, and inserted into *Nhe*I/*Xho*I and *Kpn*I/*Hind*III sites of pcDNA3.1(−) vector, respectively. The middle fragment between them was directly digested from pGD210 with *Xho*I/*Kpn*I and inserted into the corresponding site in pcDNA3.1(−). To generate BCR-ABLT315I, recombinant PCR was performed by introducing the C to T mutation into the primer and then using the obtained fragment to replace the native part between *Kpn*I/*Bcl*I site of pcDNA3.1(−)-BCR-ABL. pcDNA3.1(+)-3 × HA-Ub was a kindly gift from David Doman (Genetech, Inc., South San Francisco, CA). All constructs were verified by DNA sequencing.

For cell transfection, HEK-293T cells were transfected with TurboFect (Thermo Fisher Scientific). K562 and K562R cells were electroporated with the Cell Line Nucleofector^TM^ Kit V by using Nucleofector^TM^2b device, program T-16 (Lonza). Forty-eight hours later, cell cycle and apoptosis were analyzed by Flow cytometry and Western blot.

### Lentivirus expressing system and cell infection

FLAG-SH2 and FLAG-SH2-U-box fragment were subcloned into the *BamH* I and *Asc*I sites of the pLenti6.3-MCS-IRES2-EGFP lentiviral vector respectively. HEK-293T cells were transfected with pLenti6.3-IRES2-EGFP, pLenti6.3-SH2-IRES2-EGFP, or pLenti6.3-SH2-U-box-IRES2-EGFP together with pLP1, pLP2 and pLP/VSVG using Lipofectamine 2000 (Invitrogen) according to the manufacturer’s instructions. The lentiviral supernatants were collected after 48 hours, filtered with 0.45 μm filter (Millipore, Billerica, MA, USA). The final titer of the purified lentivirus was 1.1 × 10^9^ TU/mL. For *in vitro* cell infection and stable cell lines generation, K562 and K562R were seeded in 6-well plates and infected with 20 μL (1.1 × 10^8^ TU/mL) pLenti6.3-IRES2-EGFP (eGFP), pLenti6.3-SH2-IRES2-EGFP (SH2) or pLenti6.3-SH2-U-box-IRES2-EGFP (SH2-U-box), and twenty-four later cells were selected with 3 μg/mL blasticidin (Gibco) for 2 weeks to obtain the stable cell lines expressing eGFP, SH2 and SH2-U-box. Primary CML cells were seeded in 6-well plates and infected with 20 μL (1.1 × 10^8^ TU/mL) of pLenti6.3-SH2-IRES2-EGFP (SH2) or pLenti6.3-SH2-U-box-IRES2-EGFP (SH2-U-box). Twenty-four hours later, cells were seeded into 96-well plate and subjected to CCK8 assay. Western blot were performed 48 hours post infection.

### Antibodies and reagents

Antibodies against Bcr, p-Akt/Akt, PI3K p110β, Stat5, Erk1/2, p-Erk1/2,Bcl-xL, PARP and PathScan^®^ Bcr/Abl Activity Assay (Phospho-c-Abl, Phospho-Stat5 and Phospho-CrkL Multiplex Western Detection Cocktail) were from Cell Signaling Technology (Andover, MA, USA). Antibodies against GAPDH, β-actin and c-Abl were from Santa Cruz Biotechnology (Santa Cruz Biotechnology, CA, USA), Antibodies against FLAG or HA were from Sigma-Aldrich (St Louis, MO, USA). Secondary antibodies were chosen according to the primary antibodies origin. Proteasome inhibitor MG132 was from Calbiochem (Billerica, MA, USA). Imatinib mesylate was from Department of Hematology, Tangdu hospital, the Fourth Military Medical University. SYBR Premix Ex Taq II and Multiscript RT were purchased from TaKaRa Biotechnology (Dalian) Co., Ltd (Dalian, China).

### Western blotting, co-immunoprecipitation and ubiquitination assay

For western blotting, cells were lysed in RIPA buffer (50 nM Tris-HCl, 150 nM NaCl, 1% NP40, 0.1% SDS, 0.5% deoxycholate, 1 mM phenylmethanesulfonyl fluoride) for 30 minutes on ice. A total of 30 μg protein was separated by gradient SDS-PAGE (4–12% polyacrylamide). Proteins were then transferred to a nitrocellulose membrane. The membranes were blocked, immunoblotted with indicated primary antibodies, subsequently incubated with the corresponding horseradish peroxidase-conjugated secondary antibodies. The signals were detected with an enhanced chemiluminescence system (Tanon 5500). The band intensity of p-BCR-ABL, BCR-ABL, PI3K(P110β), p-STAT5/STAT5, pAKT/AKT, pERK/ERK and Bcl-xL was quantified by densitometry using imageJ software and normalized to β-actin, the data are the mean ± S.D. from three independent experiments.

For co-immunoprecipitation assay, FLAG-tagged SH2 or SH2-U-box was co-transfected with pcDNA3.1(−)-BCR-ABL or pcDNA3.1(−)-BCR-ABL T315I into 293 T cells and then treated with 6–10 μM MG-132 for 4−6 hours before harvest. For *in vivo* ubiquitination assay, pcDNA3.1(+)-3 × HA-Ub were also cotransfected into 293 T cells and treated as above. Protein lysates were incubated with anti-c-Abl antibody for 4 hours at 4 °C, followed by incubation with protein A + G sepharose beads overnight at 4 °C. Sepharose beads were then gently washed 3 times at 4 °C and boiled. The precipitates were resolved by 7.5% SDS-PAGE and subjected to Western blotting with anti-c-Abl, anti-Bcr, anti-FLAG or anti-HA.

### Quantitative real-time PCR (qRT-PCR) analysis

Total RNA of the the transfected cells were extracted with RNAiso Plus regent and reverse transcribed into cDNA following the instruction of Multiscript RT Kit (TaKaRa). Quantitative real-time PCR was performed with BIO-RAD C1000 Thermal Cycler according to the manufacture’s recommended protocol. The PCR primers were as follows: BCR-ABL-forward: 5′-TCCACTCAGCCACTGGAT TTAA-3′, BCR-ABL-reverse: 5′-TGAGGCTCAAAGTCAGATGCTACT-3′; GAPDH-forward:5′-TGTGTCCGTCGTCCATCTGA-3′, GAPDH-reverse: 5′-CCTGCTTCACCACCTTCTTGA-3′. qRT-PCR was performed with the following conditions: activation at 95 °C for 3 min followed by 40 cycles of 95 °C for 10 s and 55 °C for 30 s. A cycle of solubility curve was added at last to examine the amplification quality. Expression of mRNA for GAPDH was used as an internal standard.

### Cycloheximide (CHX) chase experiment

To assess the protein stability of BCR-ABL/T315I, HEK-293 T cells were cotransfected with pcDNA3.1(−)-BCR-ABL or its mutant and the pFLAG-CMV-4-SH2 or pFLAG-CMV-4-SH2-U-box. Twenty-four hours later, the cells were treated with CHX (50 μg/mL) for 0, 4, 6, 12 hours in the absence or presence of MG132 (10 μM). Then the cells were harvested and the cell lysates were subjected to Western blotting.

### Cell viability assay

Cell viability was evaluated by CCK-8 assay (Cell Counting Kit-8, Dojindo Molecular Technologies, Inc.). K562 and K562R cells stably expressing eGFP, SH2 and SH2-U-box, or primary CML cell transiently infected with SH2- or SH2-U-box-crarrying lentivirus were seeded at 300/well in sextuple in 96-well plates and incubated with/without imatinib (0.5 μM) for up to 5 days. Two hours before harvest, 10 μL of CCK-8 was added to each well and the absorbance was read on BIO-RAD iMark Microplate Reader at a wavelength of 450 nm. The experiment was repeated at least three times independently and the results were the mean ± S.D. from six replicates.

### Flow cytometry analysis of cell cycle and apoptosis

K562 and K562R cells were transiently electroporated with pFLAG-CMV-4, pFLAG-CMV-4-SH2 or pFLAG-CMV-4-SH2-U-box and 24 hours later, cells were treated with or without 0.5 μM imatinib for 24 hours. For cell cycle distribution, cells were washed with phosphate-buffered saline and fixed with ice-cold 70% ethanol for 3 hours, followed by analysis with FACScan apparatus (BD,USA). The apoptotic cells were evaluated by propidium iodine and Annexin V-FITC staining (BD, USA) and analyzed with FACScan apparatus. Early apoptotic cells were defined as PI-negative, Annexin V-positive cells. The experiment was repeated at least three times independently and the results were the mean ± S.D. from triplicates.

### Xenografts study

Four- to six-week-old nude mice and severe combined immunodeficient (SCID) mice (Shanghai Laboratory Animal Center, Shanghai, China) were housed in a dedicated pathogen-free barrier facility at the Laboratory Animal Center of the Fourth Military Medical University. K562-xenograft were established by subcutaneously injecting 1 × 10^7^ cells which were resuspended in 200 μL high concentration matrigel matrix (BD, #354248, USA) into both flanks of the nude mice. When the tumors were visible, each mouse was treated with pLenti6.3-SH2 (100 μL, 2 × 10^8^ TU/mL) at one side and pLenti6.3-SH2-U-box at the other side, via intratumoral injection every two days. K562R-xenografts were established in the severe combined immunodeficient (SCID) mice and processed in the same way. After injection, tumor size was measured every two days using a slide caliper for 16 days, and tumor volume was calculated using the following formula: volume (mm^3^) = (d^2^ × D)/2, where d and D represent the shortest and the longest tumor diameters respectively. At the end of the experiment, the animals were sacrificed and the xenografts were isolated and weighed. The data are the mean ± S.D. from the three mice. Parts of tumors were paraffin-embedded, formalin-fixed, sectioned and subjected to hematoxylin-eosin staining and immunostaining with anti-Ki-67 antibody. All of the experimental protocols in terms of cell lines and animals were carried out in accordance with the Institutional Animal Care and Use Committee guidelines and were approved by the Institutional Animal Ethics Committee of the Fourth Military Medical University (Permit No. 16001, 16002, see Supplementary information- Animal Experimental Ethical Inspection).

### Statistical analysis

Each experiment was repeated at least three times independently. Data were expressed as means ± S.D. Statistical analysis was performed with SPSS17.0 software by using Student’s *t* test (two-tailed). P value < 0.05 was defined as statistically significant.

## Additional Information

**How to cite this article**: Ru, Y. *et al*. The chimeric ubiquitin ligase SH2-U-box inhibits the growth of imatinib-sensitive and resistant CML by targeting the native and T315I-mutant BCR-ABL. *Sci. Rep.* **6**, 28352; doi: 10.1038/srep28352 (2016).

## Supplementary Material

Supplementary Figures
