# Supplementary material for: The chimeric ubiquitin ligase SH2-U-box inhibits the growth of imatinib-sensitive and resistant CML by targeting the native and T315I-mutant BCR-ABL
Source: Sci Rep. 2016 Jun 22;6:28352. doi: 10.1038/srep28352 (PMC4916441; doi:10.1038/srep28352)
Supplement: Supplementary Figures [file srep28352-s1.doc]

## Supplementary Information

**The chimeric ubiquitin ligase SH2-U-box inhibits the growth of imatinib-sensitive and resistant CML by degrading the native and T315I-mutant BCR-ABL**

Yi Ru^1^, †, Qinhao Wang^1^, †, Xiping Liu^1, 5^, †, Mei Zhang^1^, Daixing Zhong^2^, Mingxiang Ye^3^, Yuanchun Li^4^, Hua Han^1^, Libo Yao^1^, *, Xia Li^1^, *


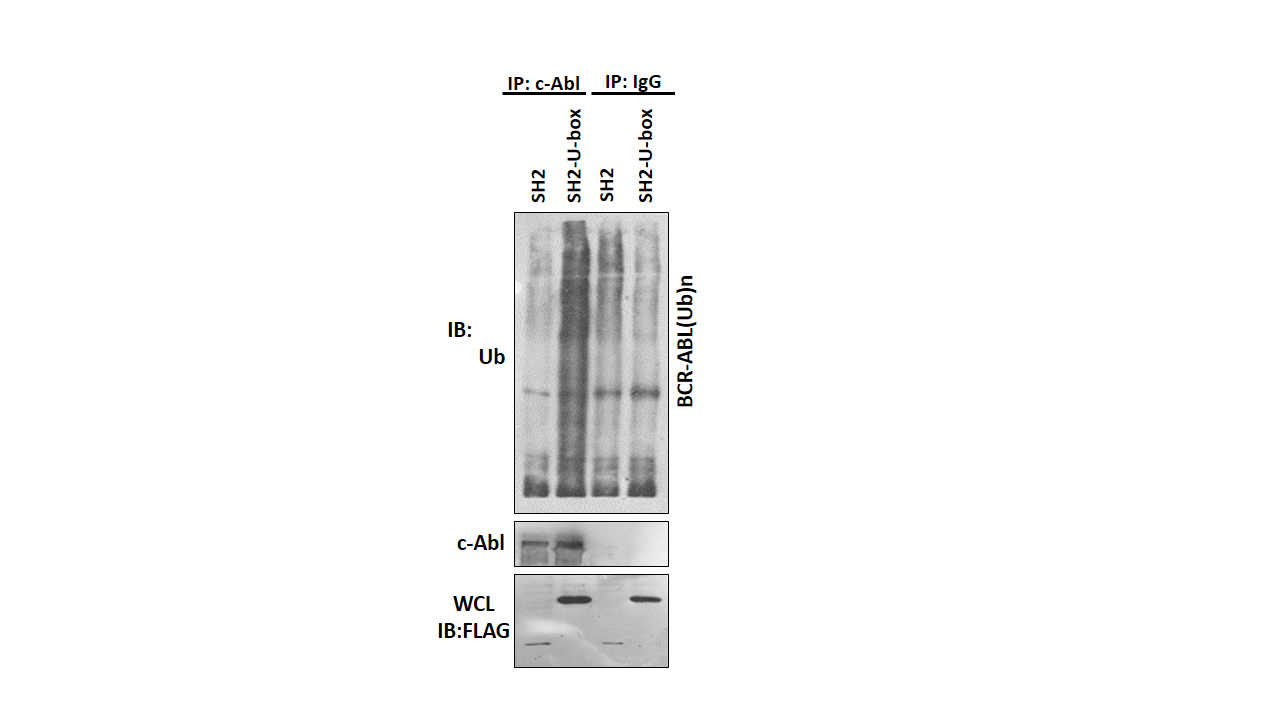


**Supplementary Figure 1. SH2-U-box promotes BCR-ABL ubiquitination.**

293T cells were transfected with the indicated constructs together with pcDNA3.1(+)-3×HA-Ub and treated with MG-132 for 4 hours. BCR-ABL ubiquitination were assessed by *in vivo* ubiquitination assay as described in Methods. Whole cell lysates (WCL) were subjected to Western blotting with anti-FLAG antibody.

**
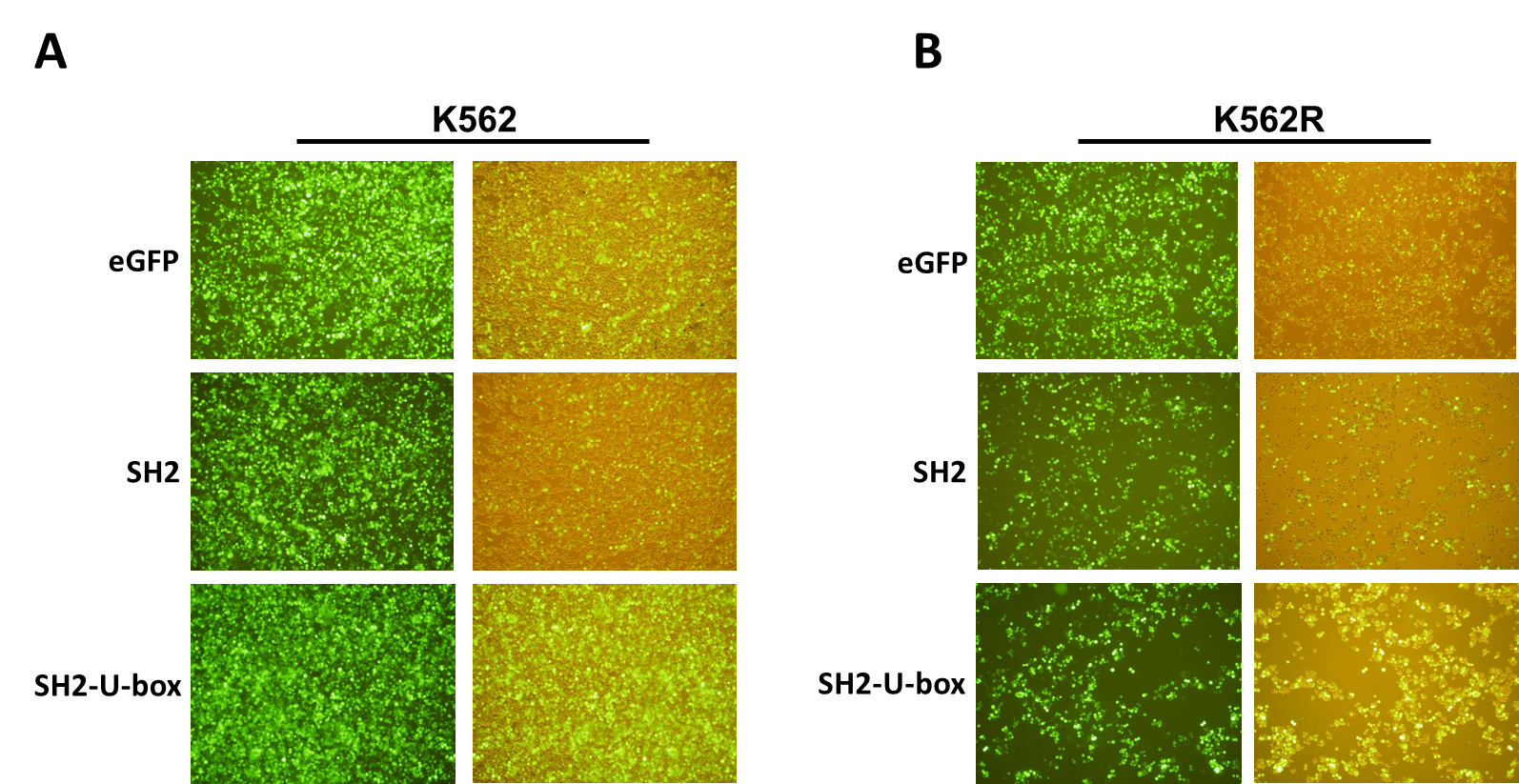
**

**Supplementary Figure 2.** K562 (A) and K562R (B) were infected with 20μL (1.1×10^8^TU/mL) pLenti6.3-IRES2-EGFP (eGFP), pLenti6.3-SH2-IRES2-EGFP (SH2) or pLenti6.3-SH2-U-box-IRES2-EGFP (SH2-U-box), and twenty-four later cells were selected with 3μg/mL blasticidin for 2 weeks. The stable cell lines were imaged using fluorescence microscopy.


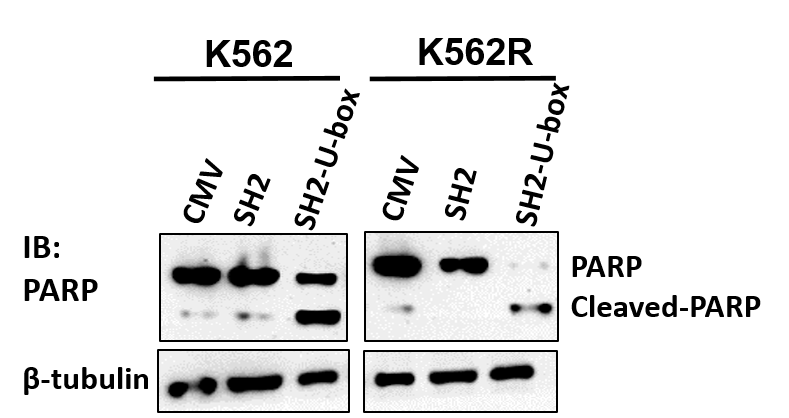


**Supplementary Figure 3. Cell apoptosis in SH2-U-box group were confirmed by PARP cleavage.** Lysate of transiently electroporated K562 and K562R cells were subjected to Western blotting with anti-PARP antibody to confirm their apoptosis status.
